# Rapamycin delays growth of Wnt-1 tumors in spite of suppression of host immunity

**DOI:** 10.1186/1471-2407-8-176

**Published:** 2008-06-21

**Authors:** Elena V Svirshchevskaya, Jacopo Mariotti, Mollie H Wright, Natalia Y Viskova, William Telford, Daniel H Fowler, Lyuba Varticovski

**Affiliations:** 1 Shemyakin-Ovchinnikov Institute of Bioorganic Chemistry RAS, Miklukho-Maklaya Street 16/10, Moscow 117997, Moscow, Russia; 2 National Cancer Institute, NIH, 37 Convent Drive, Bethesda, MD 20892, USA

## Abstract

**Background:**

Rapamycin, an inhibitor of mammalian target of Rapamycin (mTOR), is an immunosuppressive agent that has anti-proliferative effects on some tumors. However, the role of Rapamycin-induced immune suppression on tumor progression has not been examined.

**Methods:**

We developed a transplantation model for generation of mammary tumors in syngeneic recipients that can be used to address the role of the immune system on tumor progression. We examined the effect of Rapamycin on the immune system and growth of MMTV-driven Wnt-1 mammary tumors which were transplanted into irradiated and bone marrow-reconstituted, or naïve mice.

**Results:**

Rapamycin induced severe immunosuppression and significantly delayed the growth of Wnt-1 tumors. T cell depletion in spleen and thymus and reduction in T cell cytokine secretion were evident within 7 days of therapy. By day 20, splenic but not thymic T cell counts, and cytokine secretion recovered. We determined whether adoptive T cell therapy enhances the anti-cancer effect using *ex vivo *generated Rapamycin-resistant T cells. However, T cell transfer during Rapamycin therapy did not improve the outcome relative to drug therapy alone. Thus, we could not confirm that suppression of T cell immunity contributes to tumor growth in this model. Consistent with suppression of the mTOR pathway, decreased 4E-BP1, p70 S6-kinase, and S6 protein phosphorylation correlated with a decrease in Wnt-1 tumor cell proliferation.

**Conclusion:**

Rapamycin has a direct anti-tumor effect on Wnt-1 breast cancer *in vivo *that involves inhibition of the mTOR pathway at doses that also suppress host immune responses.

## Background

Breast cancer is the most frequent type of neoplasm among women accounting for almost 30% of all tumor cases. The estimated incidence in USA for 2007 is 180,000. In spite of advances in early detection and treatment, death rates have not changed significantly. Therefore, the development of new drugs for treatment of breast cancer is an area of active research. Rapamycin (sirolimus) is an antifungal antibiotic possessing immunosuppressive and anti-tumor activity by inhibiting the mTOR pathway. Rapamycin delays tumor growth in some mouse models including ErbB2 model of breast cancer [1-3]. However, 20–25% of established breast cancer cell lines are resistant to Rapamycin [4] and multiple molecular mechanisms of resistance to Rapamycin and Rapamycin-like drugs (RLD) have been proposed [5]. In addition, clinical trials involving Rapamycin or other mTOR inhibitors have shown only relatively modest responses in 7–30% of cancer patients [6-8]. Phenotypic characterization and microarray profiling of breast tumors reveal that distinct subtypes of breast carcinoma are associated with different survival rates and response to therapies. Five major groups of invasive breast carcinomas have been identified: luminal A, luminal B, HER2+/ER-, basal-like, and normal breast-like [9]. A previous report showed that transgenic mammary tumors driven by Erb-B2 are sensitive to Rapamycin [3]. Thus, a specific subset of breast cancer patients may benefit from this type of therapy.

Wnt-1 was first identified as a protooncogene activated by viral insertion in mouse mammary tumors. Transgenic expression of MMTV-regulated *Wnt-1 *gene causes extensive ductal hyperplasia and mammary adenocarcinomas in transgenic mice [10]. Although Wnt-1 itself has not been implicated in human breast neoplasms, other Wnt family members are overexpressed in human breast cancer and there is growing evidence that Wnt pathway contributes to maintenance of cancer stem cells [11]. There are no reports on the role of Rapamycin in Wnt driven mammary tumors.

Rapamycin and several RLD, such as CCI-779, RAD001, and AP23573, have been introduced into clinical trials as anti-cancer agents. These agents generally have tolerable safety profiles, although rash, nausea, leukopenia, hyperglycemia, thrombocytopenia, and depression occur in 5–70% of patients [6-8]. When evaluated as single agents, RLDs demonstrated clinical efficacy in mantle cell lymphoma (overall response rate of 38%) and glioblastoma (36%), but low response rates in locally advanced and metastatic breast (9.2%), renal cell (7%), and neuroendocrine carcinomas (5.6%) [6-8,12,13].

Mammalian TOR (mTOR) is a serine/threonine kinase involved in intracellular signaling [14]. It plays a central role in cell growth regulation by integrating signals from growth factors, nutrients, and stress events. Constitutive activation of mTOR-related messengers, including S6 kinase, eukaryotic translation initiation factor 4E-binding protein kinase (4E-BP1), and ribosomal protein S6 occurs in numerous malignancies [14-16]. mTOR plays a central role in growth regulation of immune cells, leading to severe immunosuppression, and Rapamycin is widely used for maintenance of immunosuppression in transplant patients. However, the specific effects of Rapamycin on immune cells are still not well defined.

Long lasting thymus depletion after *in vivo *Rapamycin treatment was found in mice and rats; and decreased peripheral lymphoid cells occurred only in rats [17-19]. Since mTOR plays a central role in determining the outcome of antigen recognition, Rapamycin induces anergy rather than activation of T cells [20]. In addition, Rapamycin treatment induces T regulatory cell enrichment due to the low proliferative capacity of these cells in humans [21] and mice [22], and preferentially inhibits Th1 and Tc1 cell generation as compared to type 2 T cell immune responses [23]. This immune cell dysfunction induced by Rapamycin has been proposed to accelerate tumor growth. Therefore, augmentation of specific subpopulations of immune cells through adoptive cell therapy may improve outcome in Rapamycin-treated recipients *in vivo*.

Among the many cell types which play a role in tumor eradication, type 1 CD4^+ ^Th1 and CD8^+ ^Tc1 lymphocytes (T1 cells) which secrete high levels of IFN-γ are proposed to be most relevant [24,25]. Recently, we developed ex-vivo T cell expansion protocol that permits generation of immune competent Rapamycin-resistant Th1/Tc1 (T1) or Th2/Tc2 (T2) cells [26].

In this study, we determined the anti-cancer effect of Rapamycin in Wnt-1 mouse model of breast cancer and also the effect of Rapamycin treatment on the cellular composition and function of lymphoid organs *in vivo*. We used Wnt-1 transgenic mammary tumor transplantation model that allows generation of virtually unlimited numbers of synchronous transgenic tumors in syngeneic recipients with remarkable stability of the genome [27-29]. We also examined whether adoptive transfer of Rapamycin-resistant T1 cells improves the anti-cancer effect.

## Methods

### Animals

C57BL/6 mice were purchased from Jackson Laboratory. All mice were 6–8 wk old and maintained in pathogen-free animal facility at the National Institutes of Health. All studies were conducted in an AAALAC accredited facility in compliance with the PHS *Guidelines for the Care and Use of Animals in Research*.

### Wnt-1 tumor growth and treatment in vivo

Wnt-1 tumor cells (1–2 × 10^5^) were obtained as described [27], and inoculated subcutaneously on the right flank or into the left inguinal mouse fat pad (MFP #4). The injection of cells in 50 μl of PBS was performed through the skin of anesthetized mice. Experiments were conducted either in intact non-irradiated naïve syngeneic recipients or lethally irradiated (1050 cGy) mice using a^137^Cs gamma radiation source (gamma Cell 40; Atomic Energy of Canada). Irradiated mice were reconstituted with bone marrow (5 × 10^6 ^cells/mouse) from syngeneic B6 mice administered intravenously in 200 μl of PBS. Wnt-1 cells were implanted on the same day following irradiation and bone marrow reconstitution (5 to 10 mice per group). A stock solution of Rapamycin (LP Laboratory, USA) was made in ethanol at 1 mg/ml. Mice were given daily intraperitoneal injections of 30 μg of Rapamycin in 200 μl of 0.2% carboxymethyl-cellulose (Sigma) used as a diluent. Rapamycin therapy was initiated on day 1 after tumor implantation and continued for indicated times. Control animals received injections with vehicle alone. Tumor size was measured with vernier calipers twice a week and calculated using the formula (W^2 ^× L)/2, where W and L corresponded to width and length of tumors.

### Preparation of mononuclear cells

Spleen and thymus cells were isolated by using stainless steel 40 micron wire mesh. Bone marrow (BM) was flushed from one femur and one tibia and made into single cell suspensions by passing through 25 gauge needle. Red cells were lysed by ACK buffer (Quality Biologicals, Gaithersburg, MD). Cells were washed twice in phosphate buffered saline (PBS) and transferred to complete medium (CM) consisting of RPMI 1640 (Mediatech, Herndon, VA) supplemented with 10% FCS (Gemini Bio-Products, West Sacramento, CA), pen-strep-glut, non-essential amino acids, and 2-ME 5 × 10^-5 ^M (all from Invitrogen Life Technologies, Carlsbad, CA).

### Generation of T1Rapamycin cells using CD3 and CD28 stimulation

To generate T cells that are resistant to Rapamycin, B-cells were depleted from splenocytes using goat anti-mouse magnetic particles (Polysciences, Warrington, PA). CD4 and CD8 cells were purified by CD4 enrichment kit (StemCell Technologies, USA) and cultivated separately to generate either Th1 or Tc1 cells as previously described [26]. We have included Tc1 cells which are more likely to mediate cytotoxic anti tumor responses and have persistent in vivo survival (ibid). Briefly, to obtain Rapamycin resistant T1 (1:1 Th1+Tc1) cells purified CD4^+ ^or CD8^+ ^T-cells were stimulated with CD3/CD28 beads in the presence of *N*-acetyl-cysteine (3.3 mM; Bristol-Myers Squibb, New York, NY), selective cytokines and 1 μM Rapamycin. Anti-CD3 and anti-CD28-coated beads (CD3/CD28 beads) were produced according to previously developed protocol [26] and used routinely in our laboratory at 3:1 (bead:cell) ratio. Conditioned medium was supplemented with recombinant murine IL-12 (2.5 ng/ml; R&D Systems, Minneapolis, MN), recombinant human (rh)IL-2 (20 IU/ml; National Cancer Institute (NCI)-Biologic Resource Branch (BRB) Repository), rhIL-7 (20 ng/ml; PeproTech, Rocky Hill, NJ), and anti-murine IL-4 (clone 11B.11 (10 μg/ml); NCI-BRB). Cytokine- and Rapamycin-containing medium was added on days 0, 2, and 6 to maintain 0.2–1.0 × 10^6 ^cells/ml. Addition of rmIL-12 was performed only at day 0 of T1 culture. Before injection into mice, T1 cells were analyzed by flow cytometry for purity of preparation. Seven millions of Rapamycin resistant T1 cells (T1Rapa) were injected in 200 μl of PBS intravenously into orbital sinus of mice at indicated times.

### Isolation and in vitro cultures of primary cells from Wnt-1 tumors

Tumor cell suspension was prepared as described for other organs. Briefly, tumors were excised at 1 gm of wet weight, cut into small pieces and tumor brei was prepared by pressing through 40 micron wire mesh. Single cell suspension was obtained by passing through 20–25 gauge needles. Red cells were lysed as above. Cells were washed twice in PBS and transferred into tissue culture plates (Nunc, Rochester, NY) in CM for in vitro studies. Primary cultures of tumor cells were depleted from contaminating lymphocytes by seeding cells on 100 mm culture plates in 10 ml of CM for 24–72 hours. When adherent confluent monolayer of tumor cells was formed, the plates were washed vigorously with PBS, trypsinized, and Wnt-1 cells were used for the analysis.

### Generation of Wnt-1 cell lines

Wnt-1 cells obtained from tumors as above were seeded at low density onto 90 mm tissue culture dishes. Single colonies were picked using cloning cylinders, and transferred in CM to 96-well plate. Cells were incubated until confluence and transferred to 24-well plates. Two cell lines, W1204 and W1308, with slightly different cellular morphology were used for these studies.

### Cell proliferation in vitro assay

Primary cultured Wnt-1 cells were seeded at 10^4 ^cells/well in triplicates in 96-well plates. Serial dilutions of Rapamycin at 10–0.01 mM in CM were added to the cells 24 hours later. Cells were incubated for additional 96 hours and 1 μCi 3H-thymidin (Amersham) was added for the last 4 hours. Afterwards cells were harvested (Titertek, UK), transferred onto glass filters, and dried. 3H-thymidin incorporation was estimated by beta counter (BD, USA) using scintillation liquid. As a control, intact splenocytes (5 × 10^4 ^cells/well), studied in the same way, were incubated in the presence of CD3/CD28 beads (1:1 ratio).

### Flow cytometry analysis for surface markers

For the fluorescence-activated cell sorter (FACS) analysis cells were transferred to FACS buffer (PBS, 1% bovine serum albumin, 0.05% NaN_3_). Three-color flow cytometry was performed using FACSCalibur instrument, CellQuest software (BD Biosciences) and the following antibodies: Ep-CAM-FITC, mouse anti-vimentin, rat-antimouse-FITC, anti-mouse CD3-FITC, CD4-PE, CD8-FITC, CD25-PE, CD19-PE, NK1.1-PE, CD11b-FITC, and Fas-PE (all from BD Pharmingen). Live events (5,000–10,000) were acquired with propidium iodide exclusion of dead cells.

### Analysis of apoptosis by flow cytometry

Percentage of apoptotic cell was analyzed by AnnexinV and 3,3'-dihexyloxacarbocyanine iodide (DiOC(6)) double staining. The cationic lypophilic fluorochrome DiOC(6) (Invitrogen) was used to evaluate transmembrane potential in mitochondria [30]. Splenocytes or Wnt-1 cells were harvested, washed in pre-warmed PBS supplemented with 2% of FCS, resuspended at 10^6 ^cells/ml in PBS/2%FCS and 40 μM DiOC(6) and incubated for 30 minutes at 37°C. Cells were washed with PBS/2% FCS and transferred to Annexin V binding buffer (Hepes buffer, 10 mM, pH 7.4, 150 mM NaCl, 5 mM KCl, 1 mM MgCl_2_, 1.8 mM CaCl_2_), stained with AnnexinV-APC and propidium iodide (PI), incubated in the dark for 15 min, and analyzed by flow cytometry.

### Cytokine secretion analysis

Splenocytes were harvested from control or Rapamycin treated animals at indicated times and prepared as single cell suspension as above. Cells (10^6 ^cells/ml) were plated in CM onto 24-well plates with or without CD3/CD28 beads. Supernatants were collected at 24 hours and cytokines were measured by Bio-Plex multiplex sandwich immunoassay (Bio-Rad) using Beadlyte Mouse Multi-Cytokine Beadmaster kit (Upstate, Lake Placid, NY).

### Cell cycle analysis

Cell cycle was analyzed using DAPI-stained DNA. Two million cells were harvested at indicated time, washed in ice-cold PBS, fixed by the addition of 70% ethanol and left for 2 hours at 4°C. Thereafter, the cells were washed twice in PBS, stained with 5 μg/ml of DAPI (Sigma Chemical Co) in PBS and analyzed by FACS.

### Scanning cytometry

Primary cultures of Wnt-1 cells were grown in 24-well plates (Nunc, Rochester, NY) for 48–72 hours, then washed in FACS buffer and stained with anti-mouse ep-CAM-FITC antibodies. Wnt-1 cells were analyzed by laser scanning cytometry (CompuCyte Corp., Boston, MA). The fluorescence excitation was provided by a 488 nm argon laser beam. The green fluorescence from FITC was measured using a 530/30-nm band-pass filter and amplified using a photomultiplier.

### Western blotting

After treatment with Rapamycin for indicated times, Wnt-1 primary cultured cells were washed twice with PBS and lysed in ice-cold lysis buffer (Cell Signaling Technology, Danvers, MA). Lysates were centrifuged at 12,000 × g for 10 min at 4°C, and protein concentration of the cleared cell lysates was measured using the Bio-Rad Protein Assay kit (Bio-Rad Laboratories, Hercules, CA). Thirty micrograms of protein were denatured in SDS-sample buffer, electrophoresed using 10% SDS-PAGE gels, transferred to nitrocellulose membranes, and blocked for 1 h at room temperature in TBS-T (50 mM Tris-HCl pH 7.5, 150 mM NaCl, 0.1% Tween-20) containing 5% non-fat milk. Membranes were then incubated overnight at 4°C with the indicated primary antibodies diluted 1:1000 in blocking solution. Antibodies against pp70S6K, S6K, pS6, p-Akt, and Akt were from Translational Control Sampler Kit (Cell Signaling, Beverly, MA). The appropriate secondary antibodies conjugated to horseradish peroxidase (Santa Cruz Biotechnology, CA) were used to visualize the bands (1 h incubation) with an enhanced chemiluminescence (ECL) visualization kit (Cell Signaling, Beverly, MA).

### Statistical analysis

Statistical analysis was performed using Student's t-test. Comparison values of *p *< 0.05 were considered statistically significant.

## Results

### Rapamycin delays Wnt-1 tumor growth in vivo

**Figure 1:**
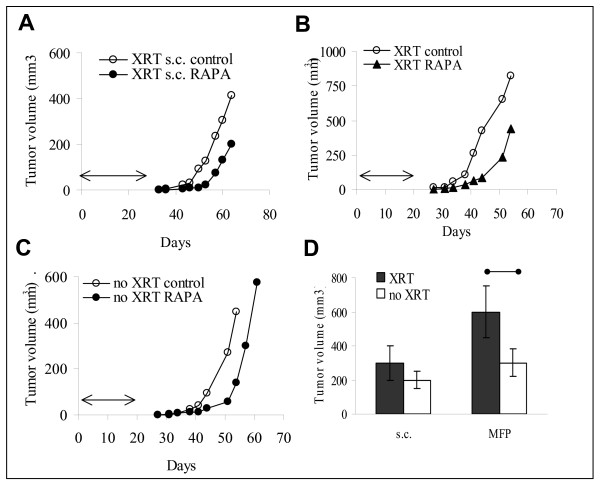
**Rapamycin induces growth delay of Wnt-1 tumors**. Growth of Wnt-1 tumors implanted subcutaneously (S.C.) (A) or into MFP (B and C) of irradiated and bone marrow reconstituted (XRT, n = 10/group) (A and B) or naïve (no XRT, n = 5/group) mice (C). D. Summary of the effect of irradiation and bone marrow reconstitution on the growth of Wnt-1 tumors implanted subcutaneously (S.C.) or into MFP. Data are presented as tumor volume at day 60 for s.c. and at day 50 for MFP after implantation. Mice were treated with 1.5 mg/kg of Rapamycin for 30 days in s.c. groups (A and D) or 20 days in MFP groups (B, C and D) starting the day after tumor implantation (arrows). Tumor size was calculated as described in Methods.

The effect of Rapamycin on growth of Wnt-1 tumors was examined in syngeneic C57BL/6 mice implanted with Wnt-1 tumor cells subcutaneously (s.c.) or into mouse fat pad #4 (MFP). For these experiments, as few as 1–2 × 10^5^cells are sufficient to generate synchronous tumors within 30 days. We used non-irradiated naïve mice or lethally irradiated and bone marrow-reconstituted animals. Rapamycin treatment for 20 days resulted in a significant delay in tumor growth evident by day 40 in naïve (non XRT) and irradiated (XRT) hosts (Fig. 1A–C). The differences in tumor growth rates between control and Rapamycin treated mice were statistically significant as determined by paired t-test. Similar results were obtained using subcutaneous (s.c.) implantation of tumor cells (10^5 ^cells/mouse) and 30 days of treatment with Rapamycin (n = 8/group, Fig. 1A and 1D).

When the effect of Rapamycin on tumor growth in non-irradiated and radiated animals was compared, it became evident that tumors grew faster in irradiated hosts. Figure 1D summarizes the results obtained on day 60 for tumors implanted s.c. and at day 50 for MFP tumors. Overall, Wnt-1 tumors grew faster in MFP than when implanted s.c. (Fig. 1D). Because growth of Wnt-1 tumors was also accelerated in irradiated mice, we hypothesized that the effect of Rapamycin could be related to its immunosuppressive action. To dissociate antitumor and immunosuppressive activities, we determined the effect of Rapamycin on Wnt-1 tumors and the immune system *in vivo *and *in vitro*.

### Rapamycin-induced suppression of immune system

**Figure 2:**
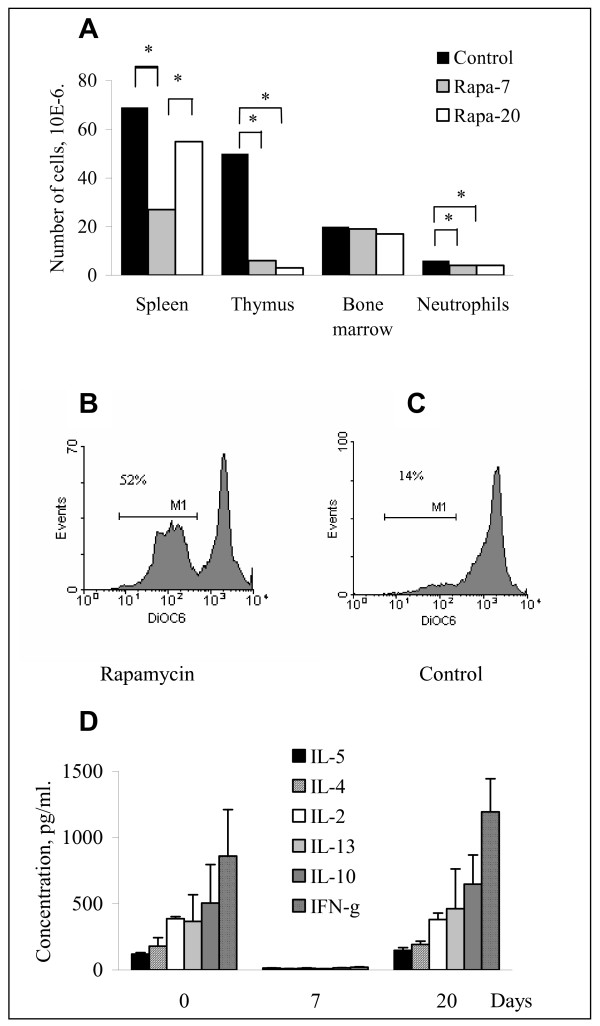
**Effect of Rapamycin on immune cells *in vivo***. A. Cell number in lymphoid organs of mice implanted with Wnt-1 tumor and treated with Rapamycin in vivo for 7 and 20 days (n = 5/group). B and C. Spontaneous apoptosis in splenocytes from mice treated for 7 days with Rapamycin (B) or control mice (C). D. Cytokine production by CD3/CD28 stimulated splenocytes from control and *in vivo *Rapamycin treated mice at days 7 and 20 post treatment.

To determine the level of immunosuppression induced by Rapamycin, lymphocytes from in vivo treated mice were analyzed at days 7 and 20 of treatment. At day 7, Rapamycin treated recipients had a substantial decrease in thymocytes and splenocytes (Fig. 2A). Although spleen cell numbers almost normalized by day 20, thymocyte counts remained severely depressed. There was no difference in the total number of bone marrow cells before and after Rapamycin treatment. Flow cytometry analysis on days 7 and 20 showed no significant difference in the proportion of splenic CD3^+^, CD4^+^, CD8^+^, CD4^+^CD25^+^, CD19^+^, NK1.1^+^, and CD11b^+ ^cells (Additional file 1), demonstrating that different subpopulations of lymphocytes are sensitive to Rapamycin to the same extent.

To determine whether Wnt-1 tumor implantation also had an effect on the immune system, an additional group of mice was treated with Rapamycin in the presence or absence of tumor. Implantation of tumors did not affect the number of cells in these groups (Additional file 1). An additional group of mice implanted with tumor cells but not treated with Rapamycin was also included. Only mice treated with Rapamycin showed a decrease in cell numbers. Thus, we concluded that immunosuppression was induced solely by Rapamycin treatment and transplantation of Wnt-1 cell did not have a detectable effect on the immune system in this model.

### Rapamycin induced apoptosis of lymphoid cells

To determine whether the decrease in splenocyte numbers found at day 7 of Rapamycin treatment was associated with apoptosis, we stained freshly isolated splenocytes from control and Rapamycin treated animals with DiOC_6_. In Rapamycin treated group, 30 to 60% of splenocytes were apoptotic as indicated by DiOC_6 _staining (Fig. 2B). In contrast, control mice had only 10 to 18% apoptotic splenocytes (Fig. 2C). Similar results with 25 to 52% of splenocytes in apoptotic fraction were obtained at day 20 of treatment with Rapamycin (data not shown).

To evaluate the function of residual lymphocytes in Rapamycin treated animals, splenocytes were harvested at day 7 and 20 of therapy and co-stimulated with CD3 and CD28 antibodies. Cytokine production was found only in CD3/28 stimulated cultures. T cell cytokine secretion was completely blocked by Rapamycin on day 7 (Fig. 2D). However, by day 20 of therapy, splenic T cell cytokine secretion recovered probably due to generation of Rapamycin resistant T cells. Rapamycin did not induce a shift away from Th1-type cytokines, since IFN-gamma production was predominant in control (day 0) and 20 day treated groups (Fig. 2D).

### Adoptive transfer of T1 cells resistant to Rapamycin did not affect Wnt-1 tumor growth

As it was shown above, Rapamycin induced apoptosis in splenocytes. On the other hand XTR also accelerated Wnt-1 tumor growth. We hypothesized that injection of Rapamycin resistant T-cells could synergize with rapamycin in tumor control. T1Rapa cells are resistant to rapamycin, while host T cells undergo apoptosis after rapamycin therapy initiation. Besides, T1Rapa cells are fully differentiated effector cells of all specificities able to execute their function immediately after the contact with specific targets. Hypothetically this could provide some advantages, i.e.: i) in case immune response to tumor antigens is possible, some of these cells would proliferate quicker than naïve T-cells; ii) tumor antigens are presented by MHC class II molecules, which mostly stimulate Th1 or Th2 responses; while Tc1 cells are more likely to mediate cytotoxic anti tumor responses; iii) these cells are rather long living as it was determined in our previous paper [26].

**Figure 3:**
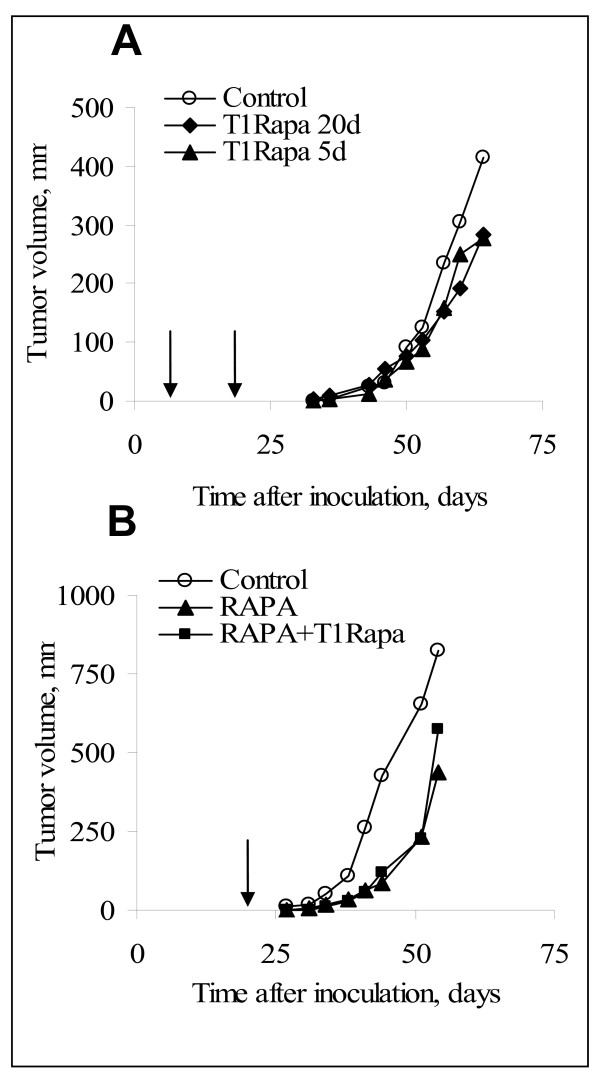
**Effects of T1Rapa cells on Wnt-1 tumor growth *in vivo***. A. Tumor growth in lethally irradiated and BM reconstituted mice implanted s.c. with Wnt-1 tumor cells (2 × 10^5^/mouse). T1Rapa cells (7 × 10^6^/mouse, 1:1 of CD4:CD8) were injected intravenously at day 5 or 20 after tumor implantation (shown with arrows). B. Tumor growth in lethally irradiated and BM reconstituted mice implanted with Wnt-1 tumor cells (10^5^/mouse) into MFP and treated with Rapamycin from day 0 to day 20. T1Rapamycin cells (7 × 10^6^/mouse, 1:1 of CD4:CD8) were injected intravenously at day 21 after tumor implantation (shown with an arrow).

To estimate the effect of Rapamycin resistant T1 cells (T1Rapa) on Wnt-1 tumor growth, irradiated and BM reconstituted mice were inoculated with tumor cells and injected either at day 5 or day 20 post transplant with 7 × 10^6 ^cells/mouse of T1Rapa cells. Adoptive transfer of T1Rapa cells did not reduce the growth of Wnt-1 tumors (Fig. 3A).

Although Rapamycin therapy delayed tumor growth, this effect was transient and tumor growth occurred after cessation of therapy. We tested whether adoptive transfer of T1Rapa cells at the end of Rapamycin treatment may delay tumor re-growth. Sequential Rapamycin therapy for 20 days followed by T1Rapa cell transfer injected on day 21 did not change Wnt-1 tumor growth as compared with Rapamycin alone (Fig. 3B). Thus, Wnt-1 tumor growth was inhibited by Rapamycin, but not by adoptive T1Rapa cell therapy.

### Direct effect of Rapamycin on Wnt-1 cells proliferation in vitro

**Figure 4:**
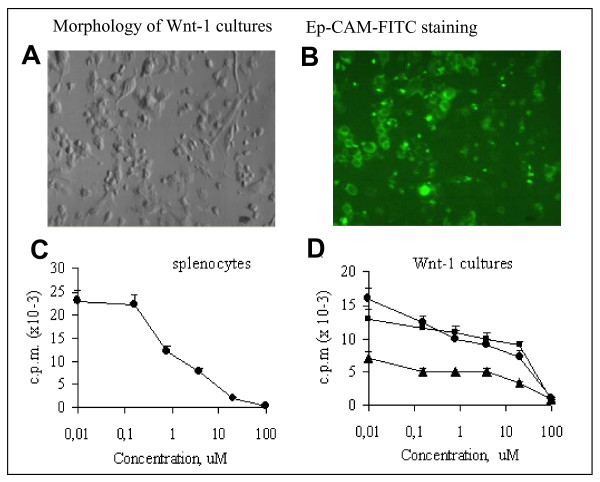
**Rapamycin inhibits Wnt-1 tumor cell proliferation**. A and B. Morphology (A) and epithelial marker Ep-CAM expression (B) in primary Wnt-1 tumor cells. C and D. Proliferation of normal CD3/28 activated splenocytes (pooled from 5 mice) (C) or primary Wnt-1 cells from 3 different tumors (D). Cells were incubated with 1 μM of Rapamycin for 72 hours and proliferation was measured by ^3^H-thymidine incorporation.

To evaluate the cellular mechanisms operational during Rapamycin induced inhibition of Wnt-1 growth we obtained purified primary tumor cells *in vitro*. Tumor cells were plated in culture medium for 2–3 days, and non-adherent cells were removed. More than 90% of the remaining adherent cells had epithelioid morphology (Fig. 4A) and were positive for epithelial cell Ep-CAM marker as determined by scanning cytometry (Fig. 4B). Additional characterization included identification of vimentin-positive myoepithelial cells which constituted less than 2% (data not shown).

The effect of Rapamycin on primary Wnt-1 tumor cell proliferation was determined *in vitro *on cells obtained from individual mouse tumors. Rapamycin inhibited proliferation of Wnt-1 cells, as well as normal lymphocytes, in a wide range of concentrations (0.1 to 50 μM), and was toxic at a concentration above 100 μM (Fig. 4C and 4D). Inhibition of Wnt-1 cell proliferation by Rapamycin was 30–50%, and growth inhibition of splenocytes was 50–90%. There was no difference in *in vitro *Rapamycin sensitivity between *in vivo *Rapa-treated or vehicle-treated cells (data not shown).

### Suppression of mTOR pathway by Rapamycin in primary Wnt-1 tumor cells

**Figure 5:**
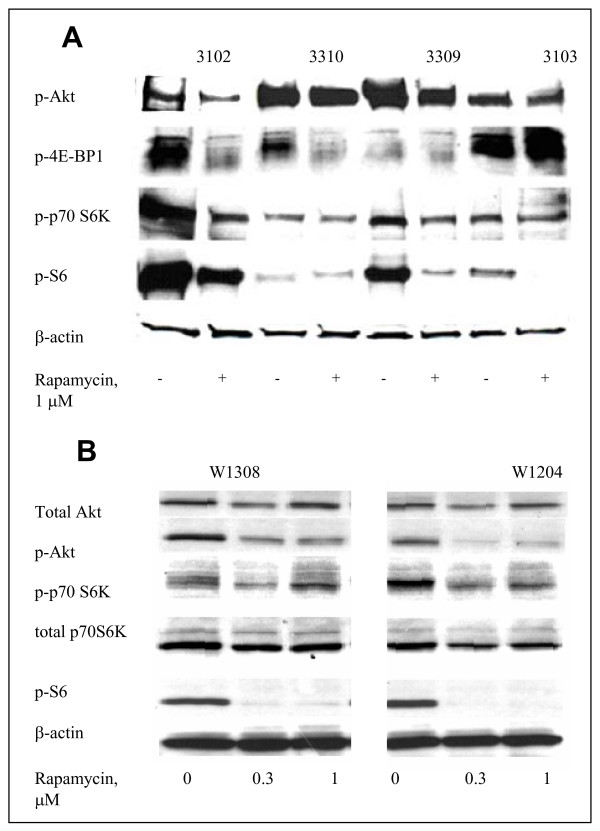
**Effect of Rapamycin on mTOR signalling in Wnt-1 cells**. A. Cells from Wnt-1 cultured for 3–4 days were trypsinyzed, seeded into flasks in complete medium and incubated overnight. Rapamycin (1 μM) was added for additional 24 hours. Western blots of cellular extracts were stained with specific antibodies to phosphorelated forms of TOR pathway messengers. B. Western blots of cell extracts from Wnt-1 cell lines, W1308 and W1204.

The effect of Rapamycin on the mTOR pathway was further examined in short-term primary cultures of Wnt-1 tumor cells and in two clonal cell lines established from these tumors. Phosphorylated Akt kinase, which activates Akt and directly phosphorylates mTOR, and expression of mTOR downstream messengers were present in all tumors, but their intensity varied in primary cells from different individual mice (Fig. 5A). Nine primary tumors were analyzed. Among others, 3 were like culture #1, and #2 last were like cultures #2 and #3, accordingly. We can see in samples #2 and #3 increased level of phosphorylated Akt kinase, while decreased amount of mTOR products. The reason for such variability is non known. This could be due to variable response of primary cells to tissue culture conditions. Phosphorylation of mTOR associated proteins was reduced by Rapamycin in 5 of 9 cultured tumors. We also generated two stable cell lines from two different primary tumors, and tested their response to Rapamycin after ten passages in vitro. Both cell lines were sensitive to Rapamycin with decreased phosphorylation of p70S6K and S6 ribosomal protein (Fig. 5B).

### Rapamycin did not induce apoptosis or cell cycle arrest in Wnt-1 cells

**Figure 6:**
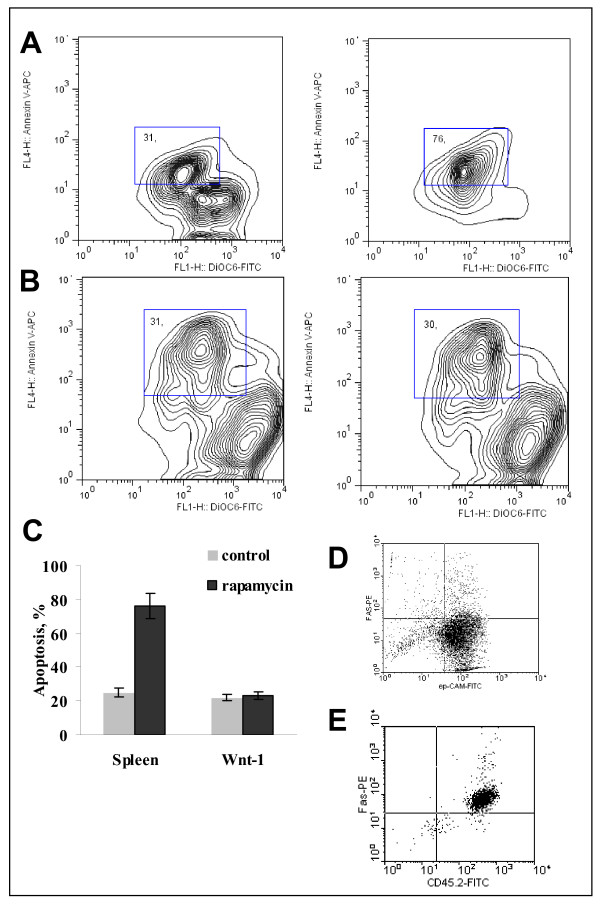
**Rapamycin induces apoptosis of splenocytes but not Wnt-1 cells**. A and B. Intact splenocytes (A) and cells from primary Wnt-1 cultures (B) were incubated with or without 1 μM Rapamycin for 24 hours, labeled with DiOC_6 _and stained with annexinV-APC. Representative contour plots for splenocytes and Wnt-1 culture are shown. Apoptotic cells are gated in a square in each panel. C. Percent of apoptotic cells (mean ± SD) is shown for 5 spleens and 6 Wnt-1 cultures. D and E. Expression of Fas on Wnt-1 cells (D) or activated splenocytes (E).

Rapamycin has been shown to inhibit the proliferation of T cells and some tumors by inducing cell cycle arrest in G1 followed by apoptosis [31,32]. We examined whether a similar process occurs in Wnt-1 tumor cells. Wnt-1 primary cultured cells (n = 6) were incubated with Rapamycin for 24 h. Freshly isolated splenocytes were used as controls (n = 5). At 24 h, nearly 30% of splenocytes and Wnt-1 cells were apoptotic in cultures exposed to media alone (Fig. 6). Rapamycin increased the percent of apoptotic splenocytes to 76% (Fig. 6A), but did not augment apoptosis of Wnt-1 cells (Fig. 6B). Fig. 6C summarizes data for Rapa-induced apoptosis in splenocytes and Wnt-1 cells.

To test whether the failure of Rapamycin to induce apoptosis in Wnt-1 cells could be due to lack of Fas expression, we examined its expression on ep-CAM^+ ^primary cultures of Wnt-1 cells. Fas expression was found in 2% to 10% of Wnt-1 cells (Fig. 6D) while in 90% of activated splenocytes (Fig. 6E). Thus, it is possible that reduced apoptotic response of Wnt-1 cells could be due to low Fas expression.

**Figure 7:**
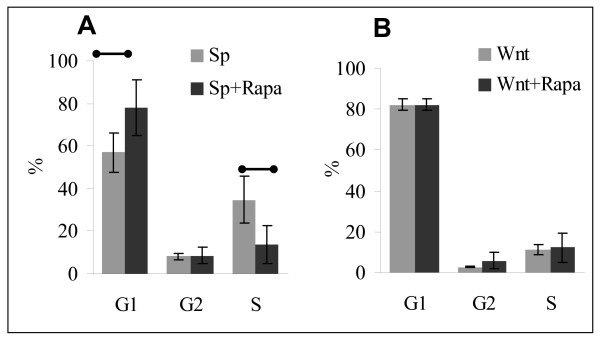
**Rapamycin induces cell cycle arrest in activated splenocytes, but not Wnt-1 cells**. Normal spleen cells stimulated with CD3/CD28 magnetic beads or primary cultures of Wnt-1 tumor cells were incubated for 72 hours with or without 1 μM of Rapamycin. Wnt-1 cells were incubated overnight to achieve adherent state before Rapamycin addition. Average percent of cells in G1, G2, and S phases from 3 independent experiments is shown. Significant differences (p < 0.02) are indicated by the bars.

We also determined the effect of Rapamycin on cell cycle progression of primary Wnt-1 cultures and CD3/28 co-stimulated splenocytes. Cells were incubated with Rapamycin for 72 hours and then collected. There was no difference between control and Rapamycin treated Wnt-1 cells, among them 80–88% of cells were found in G1 phase, and 12–18% in G2/S phase. In contrast, Rapamycin induced cell cycle arrest in activated splenic T cells where the percentage of cells in G1 phase increased from 56 to 77%, while in S phase the portion of cells decreased from 33 to 10% (Fig. 7). This result demonstrates that Rapamycin does not induce cell cycle arrest in Wnt-1 cells.

## Discussion

Several Rapamycin-like drugs have been introduced into clinical trials based on their potential antitumor effects [6-8,12,13]; however, the role of immune suppression inherent to these agents as related to their anticancer activity has not been addressed. In our study, Rapamycin induced severe immune deficiency with complete and sustained depletion of thymus, decreased numbers of immune cells in peripheral blood, transient depletion of spleen with high rate of apoptosis in mature lymphocytes, and suppressed cytokine production by T-cells within 7 days of treatment. The immune function was partially recovered on day 20 when the number of splenocytes and their ability to produce cytokines upon CD3/28 activation almost returned to normal. This was probably due to the generation of Rapamycin-resistant population of T cells. Close results were obtained in humans by Blazar B.R. and co-authors [23] who showed that rapamycin treated allogeneic BM recipients had a marked decrease in donor thoracic duct lymphocytes T cell number between days 5 and 24 post-transplant. The same study also showed that the lymphocytes had a decrease in Th1 or Tc1, but not Th2 or Tc2 cytokine production [23]. Th2 shift after in vivo rapamycin treatment was reported by several teams in humans but not in mice [26,33]. Our results in mice did not demonstrate a selective down-regulation of T1 cell function based on the profile of cytokine production. CD3/28 activated splenocytes from mice treated with Rapamycin for 20 days had comparable cytokine profiles to results in control mice.

Earlier it was shown that *in vivo *treatment of mice for 10 to 28 days with high doses of Rapamycin had no effect on myelopoiesis, as measured by BM cellularity, proliferative capacity, and number of colony-forming progenitors [34]. We also found that Rapamycin did not affect the BM cell number at day 7 or 20. This finding is rather unexpected because BM cell proliferate vigorously.

The role of T cells and especially of CD8^+ ^cytotoxic T cells in tumor surveillance has been widely studied and discussed [35,36]. In our study, Wnt-1 tumors grew slower in non-irradiated mice than in irradiated, BM reconstituted animals, suggesting that host immunity may contribute to tumor progression. Given this information, we examined the effect of Rapamycin-resistant CD8^+ ^and CD4^+ ^T-cells on Wnt-1 tumor growth *in vivo*. We used T1 cells generated *in vitro *in the presence of Rapamycin using polyclonal activation accompanied by cytokines which biased T1 differentiation, a method routinely used in our laboratory [26]. Contrary to our hypothesis, we found that the adoptive transfer of Rapamycin-resistant T1 cells did not suppress Wnt-1 tumor growth or increase the therapeutic efficacy of Rapamycin. Other T cell subsets or other immune cells, such as dendritic cells, which can be inhibited by either irradiation or rapamycin [37], play a role in tumor progression in this model. Future efforts should be directed towards evaluating alternative methods to promote immunity in the setting of rapamycin therapy.

Rapamycin and other RLD modulate G1- to S-phase progression in eukaryotic cells [38]. Rapamycin induced G1/G2 cell cycle arrest and apoptosis of activated lymphocytes, but not Wnt-1 cells *in vitro*. These results are in contrast to apoptosis induced by Rapamycin in primary adult human ALL and ErbB2 tumor cells [2,3], and indicate that inhibition of the mTOR pathway in Wnt-1 cells leads to suppression of proliferation without cell cycle arrest. These observations *in vitro *correlated with the delay of tumor growth *in vivo *which was followed by recovery after stopping the drug. Similar observations were found in ErbB2 transgenic model, with rapid re-growth of tumor after cessation of therapy [3].

Mammalian TOR forms two distinct functional complexes, termed mTOR complex 1 and 2. Previous studies indicate that Rapamycin inhibits the mTOR complex 1 pathway by blocking phosphorylation of p70 S6 kinase (S6K1) and 4E-binding protein 1 (4E-BP1), both of which are involved in protein translation and cell cycle progression [14]. In addition, prolonged exposure impairs formation of mTOR complex 2, resulting in decreased phosphorylation of Akt [39]. Previous report showed that over-expression of S6K1 and high level of phosphorylated Akt correlate with sensitivity of breast cancer cells to Rapamycin [4,16]. Rapamycin also inhibits angiogenic responses in ErbB2 transgenic mouse mammary, human hepatocellular carcinoma, and in corneal neovascularization models [3,40-42] presumably by suppression of Akt-dependent HIF-1 signaling [3]. Our data confirm that Rapamycin has a direct effect on inhibition of the mTOR pathway in Wnt-1 transgenic tumor cells in primary cultures and in cell lines derived from these tumors with suppression of proliferation and a decrease in phosphorylated forms of S6K1, ribosomal protein S6, 4E-BP1 and Akt. Additional mechanisms of Rapamycin induced MMTV-Wnt-1 transgenic tumor suppression may also play a role, including cell autophagy. Inhibition of the mTOR pathway induces macroautophagy due to deprivation of nutrients [43,44]. The transient suppression of Wnt-1 tumor growth by Rapamycin suggests that it is unlikely that these mechanisms play a significant role in this model.

Downstream components of the Wnt signaling pathway are specifically activated in a significant proportion of breast tumors [reviewed in [45]]. Activation of Wnt pathway induces expression of antiapoptotic genes in different cells which allows these cells to resist apoptosis in response to serum deprivation or induced by chemotherapeutic drugs [46,47]. Several anti-apoptotic genes, such as insulin-like growth factor (IGF) receptors, are induced by Wnt signaling and addition of IGF-I rescued MCF-7 cells from antiproliferative effects induced by Rapamycin [48]. The phosphorylation of S6K was sensitive to Rapamycin and wortmannin, a PI3K inhibitor, but resistant to U0126, a MEK inhibitor, which specifically inhibits ERK phosphorylation. Thus, Wnt signaling may partially override the effects of Rapamycin and prevent cell cycle arrest and apoptosis as shown here for Wnt-1 mammary tumor cells. In addition, Wnt directly stimulates mTOR signaling via inhibiting glycogen synthase kinase 3 (GSK3) dependent phosphorylation of tumor suppressor TSC2 [49].

## Conclusion

In conclusion, Wnt-1 mammary tumor transplanted into syngeneic hosts is a valuable model for studying the effect of immune system on cancer. Rapamycin has relatively potent direct anti-tumor effect and induces severe immune suppression that could potentially antagonize its therapeutic efficacy. However, we did not observe that adoptive T cell therapy synergizes with Rapamycin. Further studies using Rapamycin and other RLD are necessary to investigate the role of adaptive T cell transfer in other models. In addition, the evaluation of RLD in breast cancers which overexpress Wnt family members is warranted.

## Abbreviations

RLD: Rapamycin like drugs; mTOR: mammalian target of Rapamycin; XRT: irradiation; MFP: mouse fat pad; BM: bone marrow; CM: culture medium.

## Competing interests

The authors declare that they have no competing interests.

## Authors' contributions

EVS carried out in vivo studies, participated in flow, blotting analysis, cloned Wnt-1 cell lines, prepared the draft. JM helped with bio-plex assay, T1 cell generation, in vivo experiments. MHW carried out blotting, culture preparation, Wnt-1 cell cloning, proliferation assay. NYV participated in primary culture preparation, proliferation assay, blotting. WT carried out flow cytometry, apoptosis and cell cycle analysis. DHF participated in the design and coordination of the study, performed the statistical analysis, and helped to draft the manuscript. LV conceived of the study, and participated in its design and coordination and helped to draft the manuscript. All authors read and approved the final manuscript.

## Pre-publication history

The pre-publication history for this paper can be accessed here:



## Supplementary Material

Additional file 1 Table. Cell numbers and subpopulations in lymphoid organs and blood at day 7 post Wnt-1 tumor implantation with or without rapamycin treatment. The data provided represent cell numbers and subpopulations in lymphoid organs to demonstrate the effect of rapamycin treatment and not of the tumor inoculation. Click here for file
