# Supplementary material for: Rapamycin delays growth of Wnt-1 tumors in spite of suppression of host immunity
Source: BMC Cancer. 2008 Jun 21;8:176. doi: 10.1186/1471-2407-8-176 (PMC2453140; doi:10.1186/1471-2407-8-176)
Supplement: Additional file 1 — Table. Cell numbers and subpopulations in lymphoid organs and blood at day 7 post Wnt-1 tumor implantation with or without rapamycin treatment. The data provided represent cell numbers and subpopulations in lymphoid organs to demonstrate the effect of rapamycin treatment and not of the tumor inoculation. [file 1471-2407-8-176-S1.doc]

Table. Cell numbers and subpopulations in lymphoid organs and blood at day 7 post Wnt-1 tumor implantation with or without rapamycin treatment (Data are shown as mean±SD for 3 mice)

| XRT, day 0 | - | - | - | - |
| --- | --- | --- | --- | --- |
| Wnt-1, 2x10^5^, day 0 | - | + | + | - |
| Rapamycin, days 1-7 | - | - | + | + |
| Groups | 1 | 2 | 3 | 4 |
| Spleen x10^6^/organ | 72±14 | 74±12 | **17±10**^a^ | **14±8** |
| Thymus x10^6^/organ | 55±10 | 58±9 | **2.5±2.8** | **2.2±3.2** |
| Blood x10^6^ per ml | 6.3±2.1 | 5.8±2.2 | **4.5±0.5** | **4.2±0.6** |
| Bone marrow (BM) x10^6^/4 bones | 22±9 | 23±6 | 20±4 | 21±5 |
| CD3 in spleen, % | 59±6 | 62±7 | 54±5 | 57±7 |
| CD4 in spleen, % | 39±5 | 41±6 | 33±6 | 39±6 |
| CD8 in spleen, % | 20±3 | 22±2 | 19±3 | 19±2 |
| CD19 in spleen, % | 28±5 | 27±5 | 20±4 | 19±6 |
| CD11b in spleen, % | 10±2 | 11±2 | 12±3 | 9±2 |
| NK1.1 in spleen, % | 2.0±0.3 | 1.8±0.3 | 2.2±0.8 | 2.4±0.5 |

^a^ Significant differences in comparison with naïve mice are shown in bold. There were no differences between groups 1 and 2; 3 and 4.
